# Comparison of electroencephalographic changes in response to acute electrical and thermal stimuli with the tail flick and hot plate test in rats administered with opiorphin

**DOI:** 10.1186/s12883-018-1047-y

**Published:** 2018-04-19

**Authors:** Preet Singh, Kavitha Kongara, David Harding, Neil Ward, Venkata Sayoji Rao Dukkipati, Craig Johnson, Paul Chambers

**Affiliations:** 1 Massey University, Institute of Veterinary, Animal and Biomedical Sciences, Palmerston North, New Zealand; 2 Massey University, Institute of Fundamental Sciences, Palmerston North, New Zealand

**Keywords:** Opiorphin, EEG, Hot plate test, Tail flick test

## Abstract

**Background:**

The objective of this study was to compare the changes in the electroencephalogram (EEG) in response to noxious stimuli with tail flick and hot plate responses of rats administered opiorphin.

**Methods:**

Female Sprague -Dawley rats (*n* = 8 per group) randomly received intravenous (IV) injection of morphine (1 mg/kg,) or opiorphin (2 mg/kg,) or saline (0.5 ml,) in each of the three testing methods (EEG, tail flick and hot plate). Each type of test (*n* = 24 per test) was conducted in different population of rats on separate occasions. The tail flick and hot plate latencies were recorded until 5 min after test drug administration to conscious rats. The EEG was recorded in anaesthetised rats subjected to noxious thermal and electrical stimuli after test drug administration. At the end of 5 min in each of the testing methods rats were administered naloxone subcutaneously (SC) (1 mg/kg) and the test procedure was repeated.

**Results:**

There was no significant increase in the median frequency and spectral edge frequency (F50 & F95) of EEG, indicators of nociception, of morphine and opiorphin groups after noxious stimulation. Noxious stimuli caused a significant increase in both F50 and F95 of the saline group. An injection of naloxone significantly increased the F50, thus blocking the action of both opiorphin and morphine. There was a significant increase in the tail flick latency after administration of opiorphin and morphine as compared to the baseline values. Rats of morphine group spent significantly longer on the hot plate when compared to those of the opiorphin and saline groups. There was no significant difference in the hot plate latencies of opiorphin and saline groups.

**Conclusion:**

The results of this study suggest that the analgesic effect of opiorphin occurs at the spinal level and it is not as effective as morphine at supraspinal level. It may be due to rapid degradation of opiorphin or limited ability of opiorphin to cross the blood brain barrier or a higher dose of opiorphin is required for its action in the brain. Pharmacokinetic/pharmacodynamics studies along with in vivo penetration of opiorphin in the cerebrospinal fluid are required for further evaluation of opiorphin analgesia.

**Electronic supplementary material:**

The online version of this article (10.1186/s12883-018-1047-y) contains supplementary material, which is available to authorized users.

## Background

Opiorphin (QRFSR) is one of the analgesic human peptides whose effects have been investigated in rats. It has the potential to be a treatment for chronic pain including neuropathic pain [1]. Opiorphin is a penta-peptide (Gln-Arg-Phe-Ser-Arg) which has similar analgesic efficacy to morphine with fewer side effects in rats. It is a dual endogenous ectopeptidase enkephalinase inhibitor; inhibits both neutral endopeptidase (NEP) and amino-peptidase N (AP-N) [2, 3]. IC50 for NEP and APN were 8 and 30 μM, respectively [4]. These enkephalinases inactivate endogenous opioids, and blocking this inactivation has similar effects to administration of exogenous **opioids peptides** such as morphine [1, 5]. Opiorphin is produced from the PRL1 precursor in response to pain, stress and emotional state [6] both in brain and in peripheral tissue such as kidneys, digestive tract, reproductive tract (both in males and females), tears and saliva [7]. It is rapidly metabolised to RFSR-peptide by plasma exo-aminopeptidases resulting in a short half-life of only 5 min [8].

Rougeot et al. [2] demonstrated a similar analgesic efficacy of opiorphin after intravenous administration to morphine in rats using tail flick and formalin tests, but the duration of action was short. The action of opiorphin can also be prolonged by using liposomal formulations instead of conventional opiorphin formulation [1, 9]. Benyhe et al. [10] and Bogeas et al. [8] used peptidomimetic products of opiorphin which were metabolically more stable than opiorphin. The analgesic efficacy of opiorphin has been demonstrated by using “the spinally controlled thermal acute nociception, tail flick test; the supraspinally controlled mechanical acute nociception, pin pain test and the chemically-induced inflammation causing chronic pain and the formalin test [2, 3, 11]. The tail flick test works at spinal level while formalin injection test mimics chronic peripheral pain. It can be argued that both these tests do not effectively assess the brain activity and rely only on the behavioural observations. The hot plate, a supraspinally controlled thermal acute pain test has less ethical cost as compared to the formalin test. It is easy to use, highly repeatable and causes pain of only a short duration [12].

Electroencephalography (EEG) has been used to evaluate antinociception objectively in anaesthetized animals [13–15]. It is a representation of the spontaneous electrical activity of the cerebral cortex. It is well established that the cerebral cortex participates in the processing of afferent nociceptive input that results in the conscious perception of pain. Supporting evidence has been provided from human and animal studies that showed correlation of behavioural responses with EEG spectral frequency changes due to noxious stimuli [13, 14]. Changes in the EEG power spectrum, specifically, median frequency (F50), spectral edge frequency (F95) and total power of the EEG (Ptot), have been used to evaluate the anti-nociceptive efficacy of analgesics in different species of animals in response to a variety of noxious stimuli [15, 16]. To date, there have been no reports on either the effect of opiorphin on the EEG of rats subjected to noxious stimuli or the use of hot plate test to demonstrate its analgesic efficacy. A thermal supraspinal test might show different results as compared to chemical nociception induced by formalin injection.

The aim of this study was to measure and compare the changes in the electroencephalogram (EEG) in response to noxious stimuli with tail flick and hot plate responses of rats administered opiorphin or morphine.

## Methods

### Study design

This study was approved by the Massey University Animal Ethics Committee (protocols 14/86 and 15/106). Female Sprague Dawley rats (*n* = 72) were randomly selected by coin toss from a population of 500. The animals were obtained from the Small Animal Production Unit, Massey University, Palmerston North, New Zealand. Female rats were used for this study because concerns about the priapism associated with opiorphin administration in male rats [17]. The average body weight of the selected rats was 250 g. The sample size was determined by use of power calculation using ranges derived from Wisner et al. [3]. The rats were raised under standard husbandry conditions with ad libitum feed and water. The rats were kept in plastic rat cages in pairs. They were accustomed to handling for a month prior to start of the actual experiment. The person conducting the experiment was unaware of the treatment groups. The rats were randomly selected by coin toss as receiving morphine or opiorphin or normal saline.

### Tail flick and hot plate tests

The tail flick and the hot plate tests were conducted on 24 female rats for each test. These rats were divided into three groups; placebo, morphine and opiorphin, with 8 rats in each group. The drugs were injected intravenously into the tail vein. The rats were manually restrained using a perforated perspex tube exposing the tail of the rat towards the operator. The placebo group received an intravenous injection of normal saline while the morphine group and opiorphin group received an intravenous injection of morphine sulphate (10 mg/mL) at 1 mg/kg and opiorphin (0.2 mg/mL) at 2 mg/kg formulated in a solution of 100 mM PBS (55%) and 0.01 N Acetic acid (45%) [2]. Tail flick test [18] was performed by immersing the tail of the rat in hot water maintained at 55 °C, prior to and 1 and 5 min after the injection of test drugs. Tail flick reaction time at each of the time-points was recorded. Naloxone HCl (0.4 mg/mL) at 1 mg/kg was injected sub-cutaneously after the third tail flick observation. Another tail flick test was performed 5 min after the naloxone injection. The cut off time for tail flick test was 10 s.

Hot plate test [12] was performed by placing rats on a hot plate at 50 °C. Response time for observed behavioural changes like paw licking, stomping, jumping and escaping from the hot plate was recorded prior to and 1 and 5 min after the injection of test drugs. Naloxone HCl at 1 mg/kg was injected sub- cutaneously after the third hot plate test observation. A subsequent hot plate test was performed 5 min after the naloxone injection. The cut off time for the hot plate test was 15 s.

Rats used for tail flick and hot plate tests were euthanized at end of the experiment using carbon dioxide.

### EEG recording

EEG was recorded as described by Kongara et al. [16]. Briefly, rats (*n* = 24) were placed into an induction chamber and anaesthesia was induced with 2% halothane in oxygen. When the rat was unconscious, oro-tracheal intubation was performed with an 18-gauge cannula, which was connected to a T-piece breathing system. Anaesthesia was maintained with halothane in oxygen. The precision vaporizer (Fluothane; MedSource Ltd., Ashburton, NZ) was adjusted to maintain the end-tidal halothane tension (Et_Hal_) between 1.2 and 1.25%. None of the rats showed any movement responses at this minimal plane of anaesthesia (see review by Murrell and Johnson for details on minimal anaesthesia model used for recording the EEG) Ventilation was controlled using an intermittent positive pressure ventilator (V valve ventilator; Vetronics, Bioanalytical Systems Inc., West LaFayette, IN, USA) and the end-tidal carbon dioxide concentration (Et_CO2)_ was maintained between 4.7 and 6.0 kPa. Airway gases were sampled continuously from the end of the endotracheal tube connected to the breathing circuit, using an anaesthetic gas analyzer (Hewlet Packard M1025B, Hewlet Packard, Hamburg, Germany). Rats’ rectal temperature was monitored using a digital thermometer, and maintained between 37.0 and 38.5 °C with a circulating warm water blanket heating device.

Anaesthetised rats’ head was secured in a stereotaxic frame to prevent any movement of the head during EEG recording. EEG was recorded using three subcutaneous 27-gauge stainless-steel needle electrodes (Medelec, Radiometer, Auckland, New Zealand). The three electrode montage comprised of an inverting electrode over the zygomatic process of the left frontal bone, non-inverting electrode over the left mastoid process and ground electrode caudal to the occipital process. Electrode cables were connected to a custom built break-out box that was plugged into a physiological signal amplifier (Iso-Dam Signal Amplifiers; World Precision Instruments, Sarasota, FL, USA). The signals were amplified with a gain of 1000× and a band pass of 0.1–100 Hz. EEG was recorded on a Powerlab 4/20 data acquisition system (Powerlab/4sp; AD Instruments Ltd., Sydney, Australia), which digitized the signal at a rate of 1.0 kHz.

**Fig. 1:**
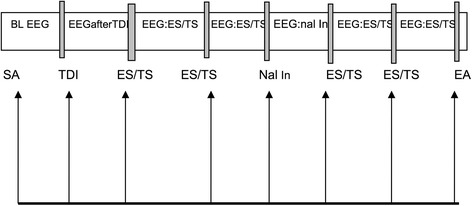
Diagram of the EEG recording pattern at different time points in rats under minimal halothane anaesthesia. SA = stabilisation of anaesthesia; BL EEG = baseline EEG; TDI = test drug injection; EEG: ES/TS = EEG recording during and after electrical or thermal stimulation ES/TS = electrical or thermal stimulation; Nal In = naloxone injection; EEG: nal In = EEG recorded after naloxone injection; EA = end of anaesthesia

EEG recording was started as soon as the rat was stabilized under anaesthesia and a 5 min baseline was recorded. The timeline for EEG recording and treatments is given in Fig. 1. At the end of the 5 min baseline, test drugs were injected. The placebo group (*n* = 8) received an intravenous injection of normal saline, while morphine (n = 8) and opiorphin (n = 8) groups received an intravenous injection of the respective drugs at 1 and 2 mg/kg. Five minutes after test drug administration and EEG recording, noxious stimuli (electrical or thermal) were applied randomly with a 10 min interval between each type of stimulus. Electrical stimulus (50 V at 50 Hz for 2 s; Valverde et al., [19]) was applied via two needle electrodes inserted to the lateral aspect of the tail base, using a Grass Stimulator (S48 K square pulse stimulator, Astro-Med Inc., Grass instrument division, Auckland, New Zealand), and thermal stimulus was applied by immersing the tail in a 55 °C hot water bath for 5 s [15].

EEG was sampled during and 5 min immediately after each type of stimulus. Naloxane HCl (1 mg/kg) was injected IV at the end of 5 min sampling after the second stimulus. Application of noxious stimuli and the EEG sampling pattern was the same as described above, after naloxane administration. Rats were euthanized at the end of the EEG recording with a halothane overdose while they were under anaesthesia.

**Fig. 2:**
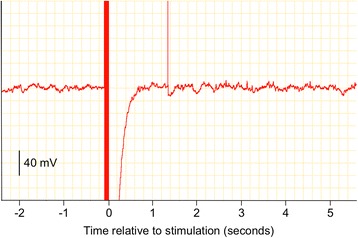
Example of raw electroencephalogram of anaesthetized rat: before and after electrical stimulation. *Note the large movement artefact during electrical stimulation

Raw EEG data were inspected visually off-line and any out-of-range data resulting from movement artefact during noxious stimulation were excluded from analysis (Fig. 2). Data were multiplied using a Welch window and fast Fourier transformation applied to each epoch, generating sequential power spectra with one-hertz frequency bins [15, 16]. The F50, F95 and Ptot were calculated for consecutive non-overlapping one-second epochs, using purpose-built software (Spectral Analyser; Craig Johnson, Massey University, Palmerston North, NZ, 2002).

### Statistical analyses

Differences in EEG variables (F50, F95 and Ptot) as well as tail flick and hot plate time, between treatment groups as well as time-points, were tested using generalised linear mixed model analysis (Littell et al. [20]) in SAS® 9.4 (SAS Institute Inc. Cary, NC, USA). The model in *proc. mixed* procedure included the fixed effects of group, time and their interaction, and random effects of animals. The covariance error structure for repeated measures over time-points, within animals in each group was determined using Akaike’s information criterion. A first-order autoregressive model was found to be the most appropriate error structure. There were significant (*P <* 0.05) between-group differences in the baseline values of all variables and hence, baseline values were included as covariates in the model. The distribution of data for different variables were tested for normality using Shapiro-Wilk, Kolmogorov-Smirnov and Anderson-Darling tests using *proc. univariate* procedure in SAS® 9.4. The residuals of data for EEG variables were found to be non-normally distributed and hence, EEG variables were normalised by Blom’s transformation employing *proc. rank* procedure in SAS® 9.4. The option of statement, NORMAL = BLOM, was used and this estimated the normal scores corresponding to the observations as per [21]. Least square means (LSM) and standard errors (SE) in original scale were used to plot graphs. Data are reported in mean ± standard errors.

## Results

### Tail flick test

**Fig. 3:**
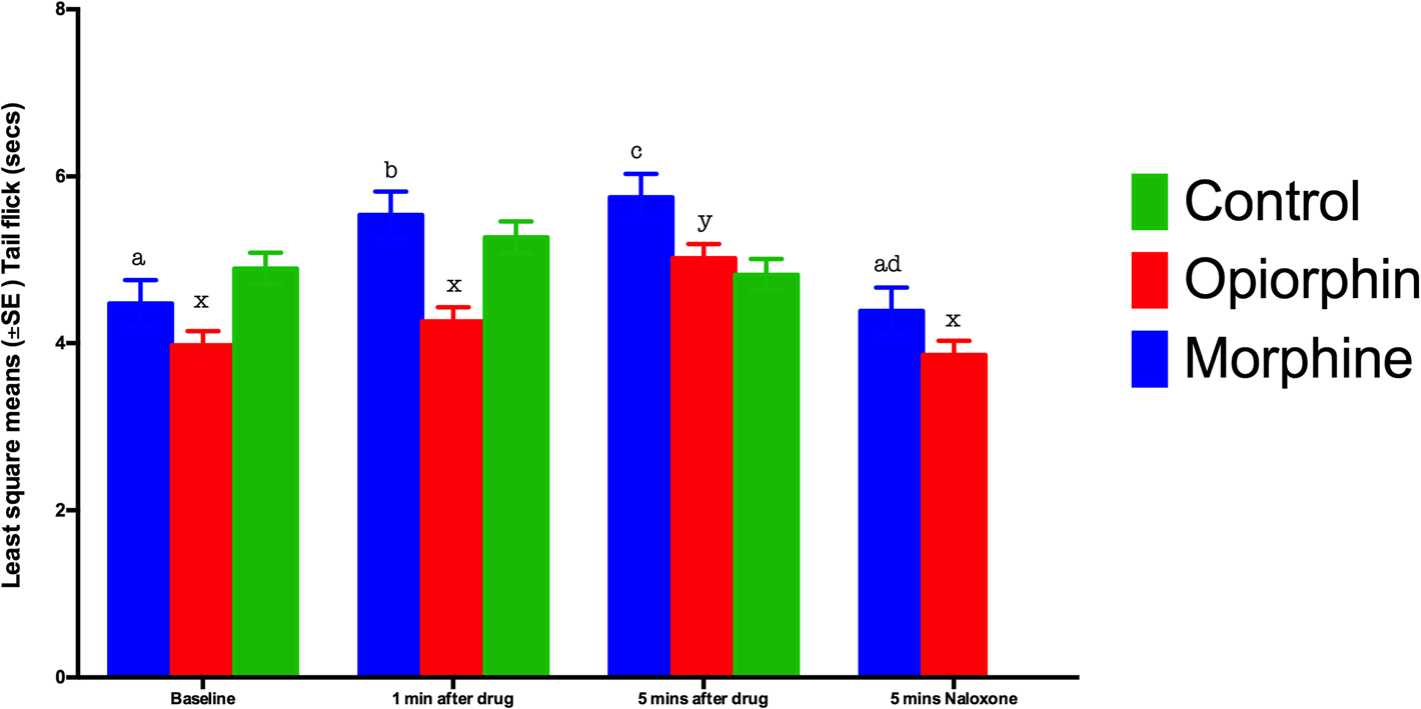
Least square means for the tail immersion time (sec) at different time points. There was a significant increase in tail flick times at one and 5 min after an IV injection of morphine and 5 min after an injection of opiorphin. There was a significant decrease in tail flick times after an IV injection of naloxone. The control group did not receive an injection of naloxone. Note 1: Mean values with an asterisk over the error bars differed significantly (p < 0.05) from their respective baseline values. Note 2: Mean values with at least one common letter over the error bars, within the each time-point, did not differ significantly (p > 0.05). Note 3: Baseline = tail flick latency before the drug administration; 1 min after drug = tail flick latency 1 min after an i.v. injection of normal saline, opiorphin or morphine; 5 mins after drug = tail flick latency 5 min after an i.v. injection of normal saline, opiorphin or morphin; 5 mins Naloxone = tail flick latency 5 min after a s.c. injection of Naloxone

Results from the tail flick test are shown in Fig. 3. There was a significant (*P* < 0.05) increase in the tail flick latencies at 5 min (5.32 s ± 0.17) after an injection of opiorphin as compared to the baseline (4.27 s ± 0.17) and this effect was blocked by an injection of naloxone (4.15 s ± 0.17). It took about 5 min for opiorphin to have its effect in the tail flick test as the tail flick latency at 1 min after the injection of opiorphin (4.56 s ± 0.17) was not significantly (*P* > 0.05) different from the baseline value. The effect of morphine on tail flick test was significant (*P* < 0.05) at 1 min after its administration (5.49 s ± 0.28) and was persistent till 5 min (5.71 s ± 0.28), after which naloxone blocked its effect (4.15 s ± 0.17).

In case of the saline group, the tail flick latencies at both time-points were non-significantly (*P* > 0.05) different from baseline reading. Comparison between groups, within a time-point, was also made. The tail flick latency at one-minute time-point in morphine group rats (5.49 s ± 0.28) was significantly (*P* < 0.05) higher than that in opiorphin group rats (4.56 s ± 0.17). Both morphine (5.71 s ± 0.28) and opiorphin (5.32 s ± 0.17) groups had significantly (*P* < 0.05) higher tail flick latencies at 5 min time-point, compared to saline group rats (4.49 secd±0.19).

### Hot plate test

**Fig. 4:**
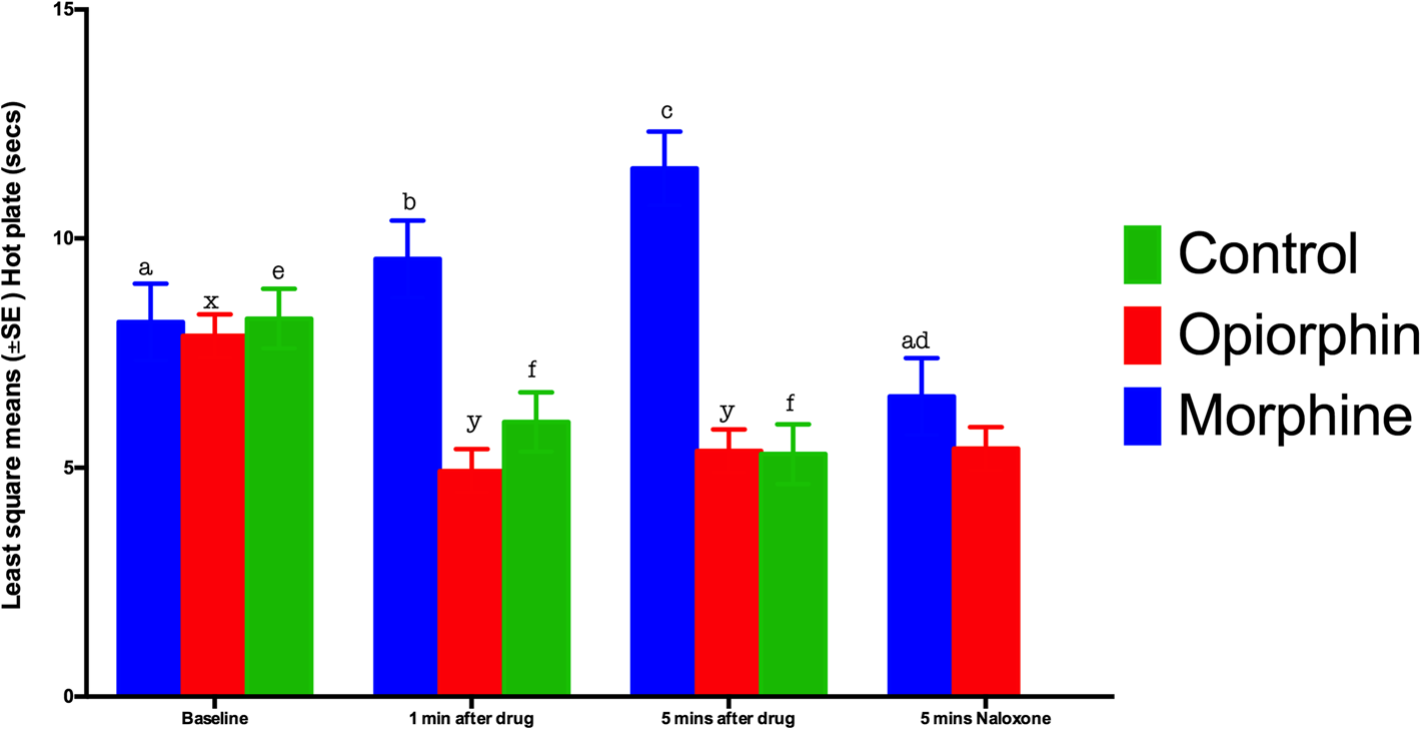
Least square means for the time spent by rats on the hot plate before and after intravenous administration of normal saline, Opiorphin and morphine. There was a significant increase in time spent by rats after an injection of morphine, while there was a significant decrease in time spent by rats after an injection of either opiorphin or normal saline. Superscripts represent significant differences. Note 1: Mean values with an asterisk over the error bars differed significantly (p < 0.05) from their respective baseline values. Note 2: Mean values with at least one common letter over the error bars, within the each time-point, did not differ significantly (p > 0.05). Note 3: Baseline = hot plate latency before the drug administration; 1 min after drug = hot plate latency 1 min after an i.v. injection of normal saline, opiorphin or morphine; 5 mins after drug = hot plate latency 5 min after an i.v. injection of normal saline, opiorphin or morphin; 5 mins Naloxone = hot plate latency 5 min after a s.c. injection of Naloxone

Figure 4 shows the results of the hot plate test. The hot plate test did not demonstrate the analgesic effects of opiorphin. There was a significant (*P* < 0.05) reduction in time spent on the hot plate in rats injected with either opiorphin or normal saline both at 1 and 5 min after injection, compared to the baseline observations. This effect was persistent after the injection of naloxone. The morphine group rats displayed typical analgesic effect. There was a significant (*P* < 0.05) increase in time spent on the hot plate at 5 after the injection of morphine. This effect was blocked by an injection of naloxone.

### EEG responses

**Fig. 5:**
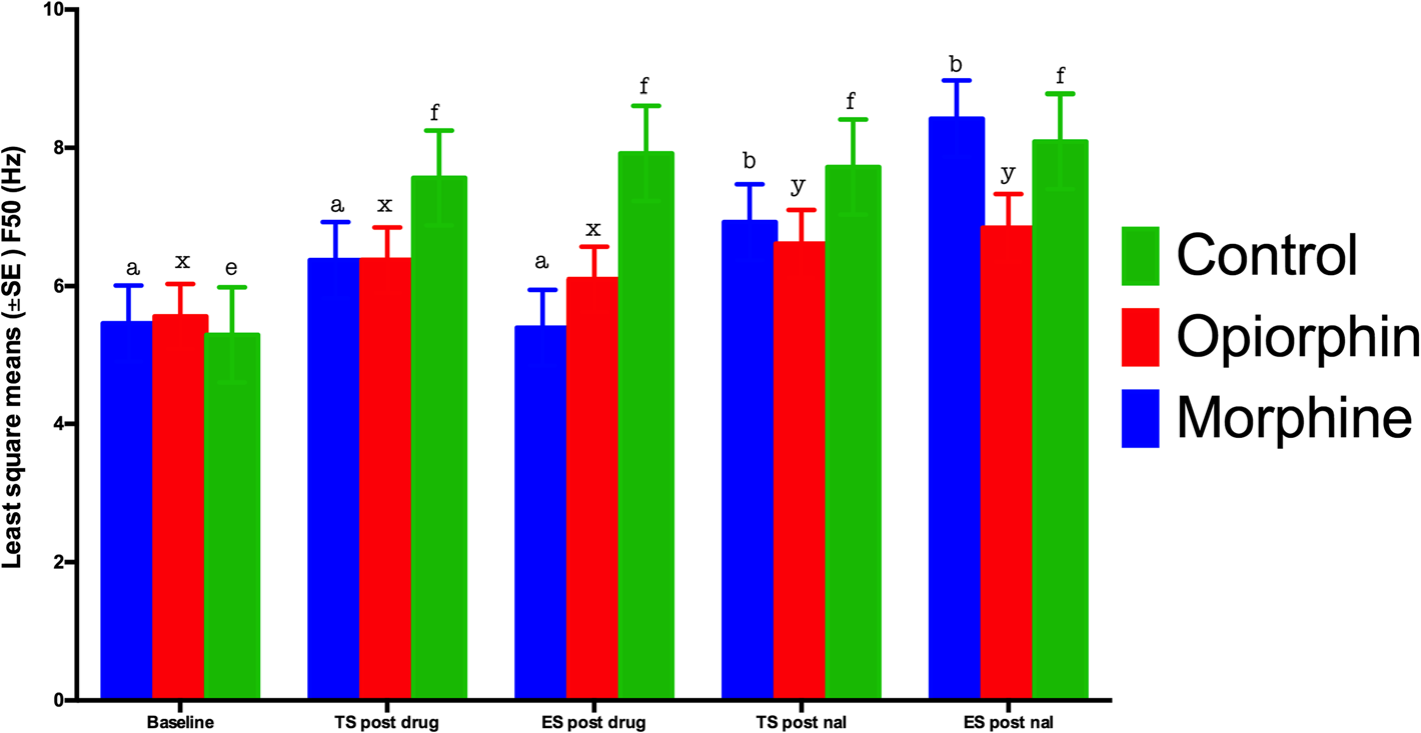
Least square means for the median frequency (F50) during thermal (TS) and electrical stimulus (ES) in rats injected with normal saline (green bars), opiorphin (red bars) and morphine (blue bars). There was a significant increase in F50 in the normal saline injected rats both during the electrical and thermal stimulus. The morphine and opiorphin effect was blocked by naloxone as there was a significant increase in F50 after administration of naloxone during the electrical stimulus. Superscripts represent significant differences. Note 1: Mean values with an asterisk over the error bars differed significantly (p < 0.05) from their respective baseline values. Note 2: Mean values with at least one common letter over the error bars, within the each time-point, did not differ significantly (p > 0.05). Note 3: Baseline = F50 before the drug administration; TS post drug = F50 after an i.v. injection of normal saline, opiorphion or morphine during thermal nociceptive stimulus; ES post drug = F50 after an i.v. injection of normal saline, opiorphin or morphine during electrical nociceptive stimulus; TS post nal = F50 after a s.c. injection of naloxone

The median frequency (F50) significantly (*P* < 0.05) increased after thermal and electrical stimulation of rats injected with normal saline as compared to the baseline readings (Fig. 5). Rats in the opiorphin and morphine groups did not show any significant (*P* > 0.05) increase in F50 compared to the baseline values. There was a significant (*P* < 0.05) increase in F50 during the thermal and electrical stimuli after the injection of naloxone in both morphine and opiorphin groups. The morphine group rats, during the electrical stimulus after naloxone injection, had significantly (*P* < 0.05) higher F50 values as compared to opiorphin group. The saline group had significantly (*P* < 0.05) higher F50 compared to morphine and opiorphin groups during the electrical stimulation.

**Fig. 6:**
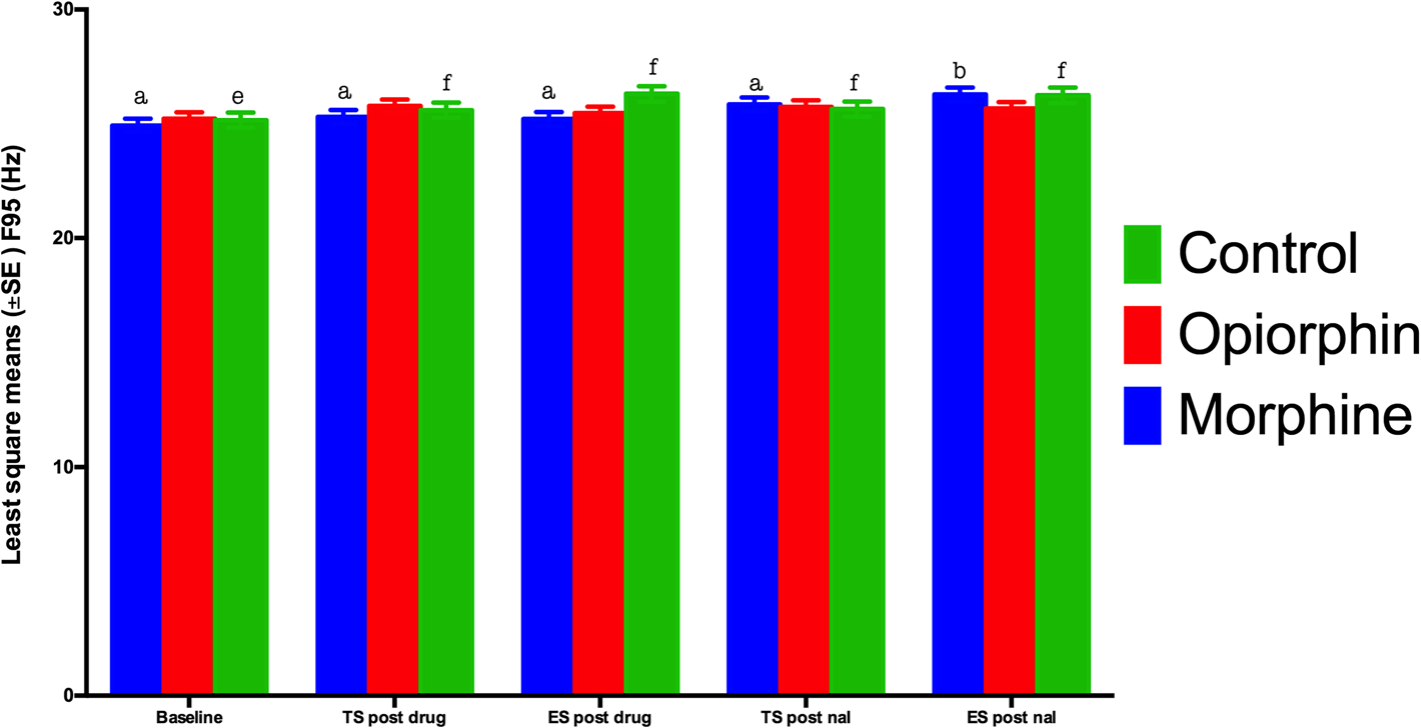
Least square means for the spectral edge frequency (F95) during thermal (TS) and electrical stimulus (ES) in rats injected with normal saline (green bars), opiorphin (red bars) and morphine (blue bars). There was a significant increase in F95 in normal saline rats. There was a significant increase in F95 post naloxone injection in morphine administered rats, both during electrical and thermal stimulus. There was no such difference observed in opiorphin group rats. Superscripts represent significant differences. Note 1: Mean values with an asterisk over the error bars differed significantly (p < 0.05) from their respective baseline values. Note 2: Mean values with at least one common letter over the error bars, within the each time-point, did not differ significantly (p > 0.05). Note 3: Baseline = F95 before the drug administration; TS post drug = F95 after an i.v. injection of normal saline, opiorphion or morphine during thermal nociceptive stimulus; ES post drug = F95 after an i.v. injection of normal saline, opiorphin or morphine during electrical nociceptive stimulus; TS post nal = F95 after a s.c. injection of naloxone

There was no significant (*P* > 0.05) difference in the spectral edge frequency (F95), compared to baseline values, following thermal and electrical stimuli in both morphine and opiorphin groups (Fig. 6). The saline group displayed significant (*P* < 0.05) increase in F95, compared to the baseline values during the electrical stimulus. The spectral edge frequency (F95) following both electrical and thermal stimuli, post- naloxone injection, significantly (*P* < 0.05) increased as compared to the baseline readings in the morphine group (Fig. 6). This increase was non-significant (*P* > 0.05) in case of opiorphin group.

**Fig. 7:**
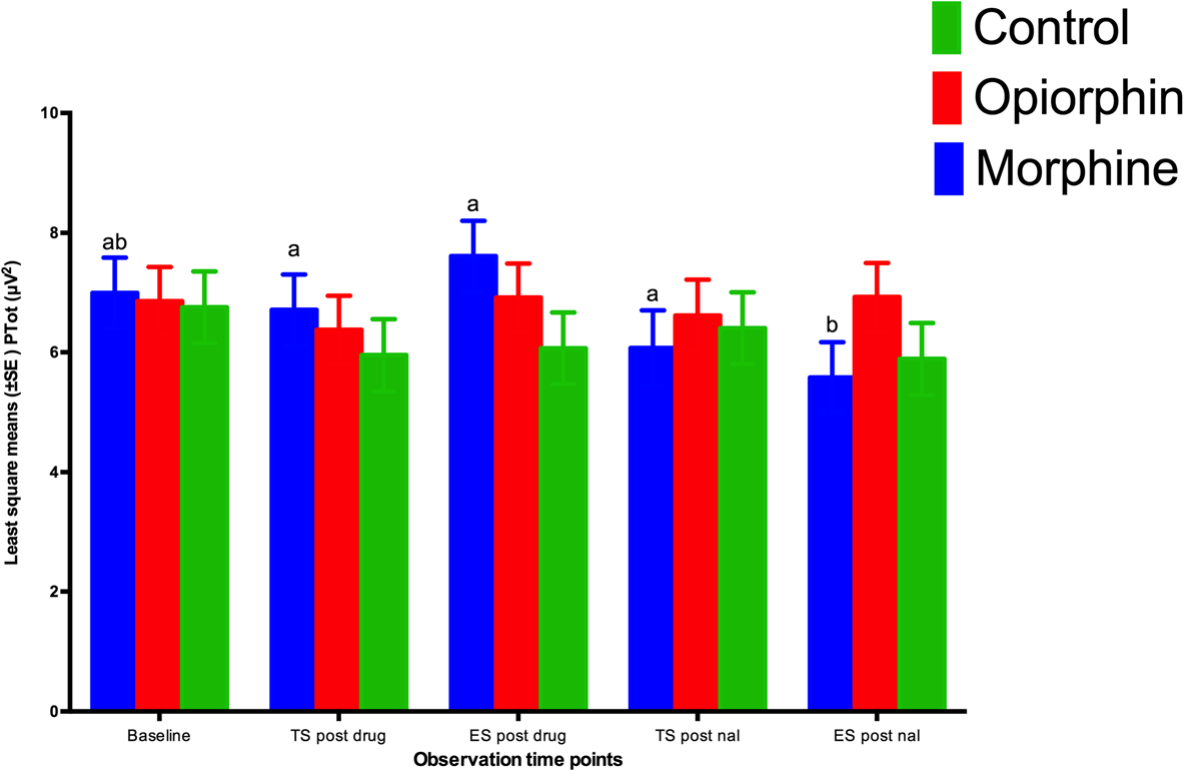
Least square means for the total power (Ptot) during thermal (TS) and electrical stimulus (ES) in rats injected with normal saline (green bars), opiorphin (red bars) and morphine (blue bars). There was no significant difference in Ptot compared to baselines in any of the three groups. The Ptot in morphine group rats significantly decreased after an injection of naloxone. Superscripts represent significant differences. Note 1: Mean values with at least one common letter over the error bars, within the each time-point, did not differ significantly (p > 0.05). Note 2: Baseline = Ptot before the drug administration; TS post drug = Ptot after an i.v. injection of normal saline, opiorphion or morphine during thermal nociceptive stimulus; ES post drug = Ptot after an i.v. injection of normal saline, opiorphin or morphine during electrical nociceptive stimulus; TS post nal = Ptot after a s.c. injection of naloxone

The total power (Ptot), compared to baseline readings, did not show any significant (*P* > 0.05) difference during both electrical and thermal stimuli after the injection of morphine or opiorphin (Fig. 7). However, after the injection of naloxone, the Ptot during electrical stimulus significantly (*P* < 0.05) decreased in morphine rats. The rats injected with morphine had significantly (*P* < 0.05) higher Ptot values during electrical stimulus as compared to the control rats.

## Discussion

The objective of this study was to compare the changes in EEG indices of nociception in response to noxious stimuli with the tail flick and hot plate latencies of rats administered opiorphin or morphine. A significant increase in F50 and F95 has been demonstrated to be the EEG responses indicating nociception after noxious stimulation of animals [16]. Studies have also demonstrated that these EEG indicators were similar to baseline during noxious stimulation after the administration of analgesic drugs. Changes in total power of the EEG may indicate a different aspect of nociception, and more related to degree of arousal during anaesthesia in animals [15]. F50 and F95 values similar to baseline after administration of either morphine or opiorphin suggests that both drugs could produce anti-nociception in response to noxious stimuli. Rats of saline group had a significant increase in F50 and F95 values compared to baseline during both electrical and thermal stimulation indicating nociception.

Following naloxone injection, F50 values in both opiorphin and morphine groups increased significantly during noxious stimulation. It is likely that naloxone displaced morphine or endogenous enkephalins from the opioid receptors and caused nociception due to noxious stimulation. Thus, opiorphin as expected functions to increase the half-life of endogenous opioids. When compared to morphine group, F50 of opiorphin group rats after an injection of naloxone during the electrical stimulus was significantly lower. The displacement of opioids by naloxone from its receptors in case of opiorphin rats was less as compared to morphine group. This could be due to continuous availability of endogenous opioids in the opiorphin administered rats that displaced naloxone from the opioid receptors. The fact that there was no significant difference in F95 after displacement of endogenous opioids in opiorphin injected rats reestablishes that naloxone may not completely block the opiorphin effect. A higher dose or continuous infusion of naloxone might be required to reverse the opiorphin effects. A continuous infusion of naloxone has been recommended to reverse the effects of the long acting potent opioid agonists [22]. The morphine group was injected only one IV bolus dose of morphine. A low dose continuous IV infusion of morphine may mimic the effects of continuous production of endogenous opioids, which was not followed in this current study.

Opiorphin caused a significant increase in tail flick latency of rats of the present study. This finding corroborates with the previously published studies in rats [2, 5]. The neurologic (reflexive) mechanisms that the tail flick test measures have been demonstrated to be spinally mediated responses to noxious stimuli [23]. This indicates the spinal analgesic effect of opiorphin in our study rats.

In this study the hot plate test was used to evaluate the supra-spinal analgesia of opiorphin, which did not show any analgesic effect of opiorphin. Wisner et al. [3] used mechanical nociceptive stimulus for assessing supraspinal analgesia after administration of opiorphin and morphine to rats, and found a significant increase in time spent on the pin areas (indicating analgesia) in both the opiorphin and morphine rats. The morphine injected rats spent higher time on the pin areas than opiorphin injected rats. Rougeot et al. [2] used formalin test as chemical induced peripheral inflammatory pain model and found significant reduction in paw flinches and body tremors after IV administration of opiorphin at 2 mg/kg. In case of morphine, a similar effect was seen, but the numbers of paw tremors was less, compared to opiorphin. Both these studies found morphine to have similar or better analgesic effect as compared to opiorphin. Opiorphin inhibits the activity of **enkephalinase enzymes**, further increasing the half-life of **endogenous opioids peptides**. The release of endogenous opioids depends on the type and intensity of the nociceptive stimulus. The endogenous opioids may not completely activate the opioids receptors. Thus, opiorphin may not exert a similar level of analgesia as compared to morphine. The present report was the only study that used hot plate test for assessing the supra-spinal effects of opiorphin. The lack of effect of opiorphin at supra-spinal level (as assessed in the hot plate test) could also be due to the reduced permeability of the blood brain barrier to the peptides [24] or a lower dose of opiorphin. Bocsik et al. [25] demonstrated that only 3% of total IV dose of opiorphin crosses the blood brain barrier as compared to 26 to 40% of IV dose of morphine. The peptides can easily cross the blood spinal cord barrier due to reduction in pericytes as compared to the blood brain barrier [26]. Thus, the blood spinal cord barrier is more permeable as compared to the blood brain barrier and that could be the reason why opiorphin exhibited significant analgesic effect in the tail flick test, but not in the hot plate test.

In this study, three methods have been used to test the efficacy of opiorphin in rats. The hot plate and the EEG results contradicted each other in opiorphin group rats. This could be due to the nature of these two tests. The EEG measures the electrical activity of the cerebral cortex. It can indirectly reflect the activity of the lower centres of the brain (e.g. brain stem areas like thalamus, PAG, RVM etc.) that have diffuse connections with the spinal nociceptive pathways. These brain stem structures have a potential regulatory influence on cortical neuronal activity especially during periods of unconsciousness and anaesthesia [27, 28]. In the current study, the EEG has been recorded in anaesthetized rats. It is likely that the anti-nociceptive effect of the opiorphin demonstrated in EEG responses, might be due to its potent effect on spinal nociceptive mechanisms that has been reflected indirectly through diffuse connections with the cortical neurons. It is also possible that opiorphin might have a weak anti-nociceptive effect at supra spinal level due to its low permeability to blood brain barrier. The EEG could represent the combined effect of opiorphin at both spinal and supra spinal levels inhibiting changes in F50 and F95. Other objective (experimental) methods such as measuring the changes in electromyographic reflex thresholds [29] and expression of the neural marker of nociception, *c*-*fos* [30, 31] would further delineate the neural basis of the antinociceptive effects of opiorphin. In this study, only one dose of opiorphin (2 mg/kg) was tested based on the dose response curves previously reported [2, 3]. A dose response curve of opiorphin using the hot plate test should be conducted for further evaluation of supraspinal analgesia due to opiorphin administration. The hot plate test is a behavioural test in conscious subjects. The behavioural endpoints in rats associated with the hot plate test are complex and difficult to interpret which adds variability to the outcome of the test, as can learning. EEG is a more sensitive test as compared to the hot plate test.

One important factor that may influence the thermal test latencies in this study is the sex of the study rats. Previous studies have demonstrated the differences in pain perception between male and female rats and mice in a formalin induced pain model [32, 33]. Intact females showed significantly more nociceptive responses than intact males. In contrast, the influence of sex difference on hot plate latencies has been reported to be non-significant in rats [12]. In female rats, the stage of oestrous cycle may also affect the behavioural responses to nociceptive pressure stimuli [34], and the hot plate latencies [35]. When the rats were tested during the oestrus phase of the cycle a significant reduction in latencies was detected. In mice, the influence of stage of oestrous cycle on responses to formalin induced nociception was reported to be non-significant [32]. Based on the variable findings of the previous studies in rats and mice it appears that the influence of the sex and stage of the oestrous cycle on pain responses is dependent on the type of noxious stimulation and animal. It is likely that the thermal latencies reported in the female rats of the present study might differ in male rats but the significance of the difference is unknown.

## Conclusion

There are several dual enkephalinase inhibitors currently under clinical trials show promising results in their preclinical studies [1]; especially orally administered P37 [36] which has potential to replace opioid pain management protocols in diabetic neuropathy***.***

Further research is required to enable to use opiorphin for clinical pain management. There is a need to conduct a proper pharmacokinetic study to know the half-life, distribution and elimination of opiorphin in the target species. The rapid breakdown of opiorphin peptide could be a hindrance in testing its analgesic efficacy. A different drug delivery approach may be required to make it a viable candidate for a new class of analgesic drugs.

## Additional files

Additional file 1: SAS output OPOR rats. Description: SAS output file for statistical analysis of EEG data acquired during this study. (MHT 280 kb)

Additional file 2: Tail flick and hot plate results. Description: SAS output file for statistical analysis of tail flick and hot plate data acquired during this study. (MHT 145 kb)

## Abbreviations

AP-N: Amino-peptidase N
EEG: Electroencephalogram
F50: Median Frequency
F95: Spectral edge frequency
IV: Intravenous
NEP: Endopeptidase
PBS: Phosphate buffer saline
Ptot: Total power
SC: Subcutaneous
